# The Aldosterone–Immune Axis in Primary Aldosteronism: Linking Adrenal Pathogenesis to Systemic Inflammation and Target-Organ Damage

**DOI:** 10.3390/biomedicines14102321

**Published:** 2026-10-13

**Authors:** Noorzaileen Eileena Zaidi, Elena Aisha Azizan, Adli Ali

**Affiliations:** 1 Department of Medicine, Faculty of Medicine, Universiti Kebangsaan Malaysia (UKM), Kuala Lumpur 56000, Malaysia; noorzaileen@ukm.edu.my (N.E.Z.); elena.azizan@ukm.edu.my (E.A.A.); 2 Research Center, Hospital Tunku Ampuan Besar Tuanku Aishah Rohani, Universiti Kebangsaan Malaysia Specialist Children’s Hospital, Kuala Lumpur 56000, Malaysia; 3 Department of Paediatrics, Faculty of Medicine, The National University of Malaysia, Kuala Lumpur 56000, Malaysia

**Keywords:** aldosterone, primary aldosteronism, mineralocorticoid receptor, adrenal pathogenesis, systemic inflammation, target-organ damage, hypertensive

## Abstract

Primary aldosteronism (PA) is the most common endocrine cause of secondary hypertension and is increasingly recognised as a systemic disorder extending beyond haemodynamic dysregulation. Accumulating evidence suggests that chronic autonomous aldosterone excess promotes maladaptive immune activation, establishing an aldosterone–immune axis that may contribute to cardiovascular, renal, and metabolic injury independently of blood pressure elevation. In some patients, glucocorticoid co-secretion may further amplify tissue-specific mineralocorticoid receptor (MR) signalling. Human and experimental studies indicate that aldosterone modulates both innate and adaptive immunity. Patients with PA demonstrate altered monocyte transcriptional profiles, enhanced vascular inflammatory activity, and increased pro-inflammatory cytokine signalling in monocyte-derived macrophages compared with blood-pressure-matched essential hypertension controls. Experimental models further show that sustained aldosterone exposure promotes macrophage M1 polarisation, dendritic cell activation, Th17 expansion, and regulatory T-cell suppression. These inflammatory changes are accompanied by oxidative stress, endothelial dysfunction, fibroblast activation, and extracellular matrix remodelling across cardiovascular and renal tissues. Emerging evidence is also consistent with a role for trained innate immunity, whereby prolonged aldosterone exposure may induce persistent epigenetic and metabolic reprogramming of monocytes and macrophages. Whether such reprogramming persists after biochemical correction by adrenalectomy or MR antagonism in human PA has not yet been directly demonstrated and remains an important open question. If confirmed, these mechanisms may help explain the partial irreversibility of target-organ damage despite successful treatment. This review synthesises current evidence linking adrenal somatic pathogenesis to systemic immune dysregulation and target-organ injury in PA. We propose that PA should be viewed not solely as a hypertensive disorder, but as a chronic fibro-inflammatory endocrine disease with important implications for early diagnosis, risk stratification, and targeted therapy. Future prospective human studies are needed to confirm the mechanistic hierarchy proposed and to identify the immunological targets most amenable to therapeutic intervention.

## 1. Introduction

The modern understanding of primary aldosteronism (PA) rests on two converging discoveries in the early 1950s. In 1952, Tait, Simpson, and Grundy characterised the effects of a previously unidentified adrenocortical hormone on renal ion and fluid homeostasis [1]; its chemical structure was resolved two years later in collaboration with Tadeus Reichstein, formally establishing aldosterone as the principal mineralocorticoid [2,3,4]. Aldosterone, synthesised exclusively in the zona glomerulosa (ZG) under the convergent regulation of the renin–angiotensin–aldosterone system (RAAS) [5,6,7,8,9], adrenocorticotropin [10], and extracellular potassium ion (K^+^) elevation [11,12]. Jerome Conn’s original description in 1955 of an aldosterone-secreting adrenocortical adenoma presenting with hypertension, hypokalaemia, and suppressed plasma renin activity [13,14,15] anchored the condition within a narrow diagnostic framework that persisted, largely unchallenged, for three decades. The prevailing assumption that hypokalaemia was a diagnostic prerequisite proved both influential and wrong. Normokalaemia characterises most confirmed cases [16], and the misinterpretation of a normal serum potassium as evidence against the diagnosis has become the primary driver of a diagnostic delay that, in international cohort data, extends to years and exposes patients to preventable and partially irreversible organ injury [17,18]. Contemporary epidemiological data have rendered that estimate untenable. PA is now established as the most common specifically treatable aetiology of secondary and treatment-resistant hypertension [19,20,21,22], affecting an estimated 5 to 10% of all hypertensive patients and a substantially higher proportion of those with resistant disease, despite the availability of the aldosterone-to-renin ratio as a validated first-line biochemical screen [23].

The delayed diagnosis of PA carries consequences that extend well beyond uncontrolled hypertension. Compared with blood-pressure-matched patients with essential hypertension, PA confers significantly higher rates of stroke, non-fatal myocardial infarction, and atrial fibrillation (AF), with odds ratios of 4.2, 6.5, and 12.1, respectively, as established by Milliez and colleagues in a landmark matched case–control analysis [24,25]. A systematic review and meta-analysis confirmed the persistence of this excess event burden across multiple independent international cohorts, independent of systemic blood pressure [25]. Structural cardiac injury follows the same pattern: left ventricular remodelling in PA consistently exceeds that observed in cohorts matched for blood pressure, hypertension duration, and treatment intensity [25,26,27,28], implicating direct aldosterone-mediated myocardial effects that operate independently of haemodynamic load. These findings establish that the excess cardiovascular burden of PA extends beyond haemodynamic mechanisms, and has motivated investigation of an aldosterone–immune axis linking adrenal pathogenesis to systemic inflammation and target-organ damage.

Reconceptualising PA as a systemic inflammatory disorder requires recognising that both MR expression and aldosterone signalling extend well beyond the renal tubular compartment. In classical epithelial target tissues, aldosterone specificity is conferred pre-receptorally by 11β-HSD2, which inactivates cortisol to receptor-inactive cortisone, preventing glucocorticoid occupancy of the MR despite a 100-fold to 1000-fold molar excess of circulating cortisol [29,30]. In non-epithelial tissues lacking 11β-HSD2, the MR is constitutively accessible to both aldosterone and glucocorticoids [31]. Transcriptomic and immunohistochemical studies have confirmed MR expression across innate and adaptive immune lineages, cardiomyocytes, endothelial cells, VSMCs, adipocytes, and central neurones [32,33,34,35,36,37,38,39,40], mapping the potential reach of continuous aldosterone excess onto every major organ system implicated in PA morbidity. Randomised trial evidence supports that MR antagonism reduces cardiovascular mortality beyond what haemodynamic mechanisms alone can explain [41,42].

The mechanistic architecture of this aldosterone–immune axis originates within the somatic genomic pathology of the adrenal gland. Structurally divergent mutations in *KCNJ5*, *ATP1A1*, *ATP2B3*, and *CACNA1D* subvert ZG calcium homeostasis through distinct ionic mechanisms yet converge upon a single pathological output: constitutive *CYP11B2* transcription and renin-independent aldosterone hypersecretion [43,44,45,46,47,48,49]. This genomic convergence, however, conceals meaningful biological heterogeneity. *KCNJ5*-mutant adenomas, which predominate in East Asian female patients and produce higher absolute aldosterone excess, may carry distinct downstream inflammatory profiles relative to *CACNA1D*-mutant disease [45,46], and their conflation within unselected PA cohorts’ risks masking subtype-specific immunological differences of direct mechanistic and therapeutic relevance. The axis is further distinguished by its bidirectionality at the anatomical source: adrenal mast cells promote ZG proliferation and steroidogenesis through serotonin-mediated paracrine signalling [50], suggesting that the adrenal immune microenvironment may act as an amplifier of autonomous secretion; however, the quantitative contribution of intra-adrenal immune signalling to the peripheral inflammatory phenotype of human PA has not been directly established [51].

That this autonomous secretion propagates a measurable systemic inflammatory state has been directly demonstrated in human PA. Patients exhibit increased arterial wall metabolic activity on 18F-FDG positron emission tomography, a reprogrammed monocyte transcriptome, elevated TNF-α expression in monocyte-derived macrophages, and a Th17/Treg imbalance not replicated in blood-pressure-matched resistant hypertension controls, collectively supporting aldosterone excess, rather than elevated blood pressure, as a principal driver of immune dysregulation [52,53,54]. Whether this inflammatory phenotype reflects durable epigenetic reprogramming of innate immune cells consistent with the trained immunity paradigm, and whether any such reprogramming persists after biochemical correction of PA by adrenalectomy or MR antagonism, remain unresolved questions of direct clinical relevance. Macrophage-mediated oxidative stress, endothelial dysfunction, and pro-fibrotic signalling may propagate local tissue injury through haematopoietic and neuroendocrine pathways that outlast the hormonal excess that initiated them, contributing to the partial irreversibility of target-organ damage characteristic of delayed PA diagnosis.

In this review, we synthesise evidence for the aldosterone–immune axis as a mechanistic framework linking adrenal somatic pathogenesis to systemic inflammation and hypertensive target-organ damage. We examine MR signalling across immune and cardiovascular tissues, innate and adaptive immune activation, the trained immunity hypothesis, and the downstream pathways driving cardiovascular and renal structural remodelling. Throughout, findings established in human PA cohorts are explicitly distinguished from experimental and *in vitro* evidence, and mechanistic hypotheses not yet confirmed in human disease are identified as such. We conclude by examining the therapeutic implications of reconceptualising PA as a chronic fibro-inflammatory endocrine disorder, with particular attention to the translational priorities that prospective human studies must address to convert this mechanistic framework into clinically actionable targets.

## 2. Materials and Methods

This narrative review was informed by a comprehensive literature search of the PubMed, Scopus, and Web of Science databases from database inception to June 2026. The search strategy incorporated combinations of the following key terms: “primary aldosteronism”, “aldosterone”, “renin–angiotensin–aldosterone system (RAAS)”, “mineralocorticoid receptor”, “trained immunity”, “endothelial dysfunction”, “cardiac fibrosis”, “renal injury”, and “target-organ damage”. Eligible studies included original mechanistic investigations involving human PA cohorts, experimental animal models of aldosterone excess, or *in vitro* human cell systems that examined the aldosterone–immune axis, immune-mediated mechanisms, or downstream target-organ injury. Particular emphasis was placed on seminal studies and recent multi-omic investigations, including genomic, transcriptomic, single-cell, single-nucleus, and spatial transcriptomic analyses that have advanced understanding of the immunobiology of aldosterone-producing adenomas. Review articles, editorials, and expert consensus statements were consulted to provide background and contextual interpretation but were not cited as primary sources of mechanistic evidence. Studies were selected based on their scientific relevance, methodological quality, and contribution to the evolving understanding of immune dysregulation in PA.

## 3. Adrenal Pathogenesis: Genomic and Somatic Drivers of Autonomy

The aldosterone–immune axis originates at the level of adrenal cortical pathology, and its systemic immunological consequences require an understanding of how physiological, stimulus-coupled mineralocorticoid secretion is transformed into the continuous, feedback-refractory aldosterone excess that defines PA. Three mechanistic domains constitute this foundation. Physiological *CYP11B2* transcription in ZG is an intrinsically self-limiting, calcium-dependent process whose temporal architecture actively constrains mineralocorticoid output between secretory episodes. Somatic driver mutations in APAs dissolve this constraint entirely, imposing constitutive intracellular calcium elevation through structurally divergent but functionally convergent ionic mechanisms. APCCs extend autonomous steroidogenic activity below the threshold of macroscopic adenoma formation, providing a cellular basis for the bilateral and CT-negative disease spectrum that discrete tumour biology cannot explain. Together, these domains establish PA not as a single genomic entity but as a convergent calcium channelopathy operating across a continuous morphological spectrum [22,55], generating the continuous, non-pulsatile mineralocorticoid excess that constitutes the necessary upstream precondition for the systemic immune dysregulation examined in subsequent sections [25,56].

Interpreting the mechanistic evidence presented in this review requires recognition of the substantial biological heterogeneity underlying PA. The recurrent somatic driver mutations associated with APAs may not be immunologically equivalent; *KCNJ5*, *CACNA1D*, *ATP1A1*, and *ATP2B3* mutations each give rise to distinct aldosterone secretory profiles [46,47,48,49,57,58,59], tumour microenvironments [53], and transcriptomic landscapes [60,61], that are likely to shape immune responses in different ways. Consequently, analyses that combine these molecular subtypes may obscure biologically and therapeutically relevant mechanisms. Throughout this review, findings derived from genotype-specific cohorts or experimental models are explicitly identified where possible, whereas studies involving unselected PA populations are interpreted accordingly. Further genotype-stratified mechanistic investigations will be essential to define how individual driver mutations influence immune remodelling, tumour evolution, and therapeutic response [60,61,62,63].

### 3.1. Physiological Regulation of Mineralocorticoid Biosynthesis

**Figure 1:**
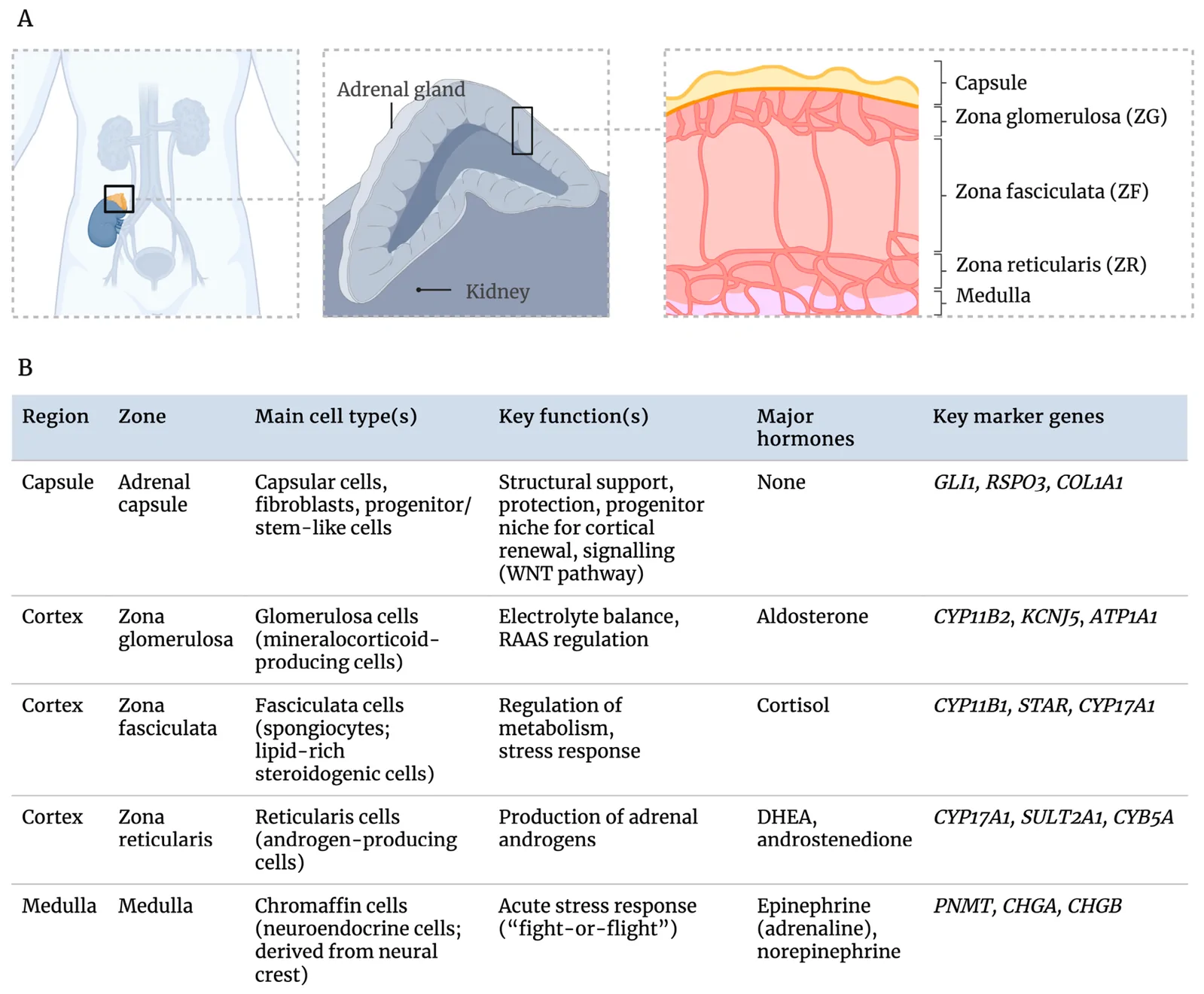
Anatomy and functional zonation of the human adrenal gland. (**A**) Anatomical localisation of the adrenal gland superior to the kidney (left), with a cross-sectional representation showing the outer cortex and inner medulla (middle). A histological schematic (right) illustrates the fibrous capsule and the three cortical layers: zona glomerulosa, zona fasciculata, and zona reticularis. (**B**) Summary of adrenal compartmentalisation showing the principal cell types, physiological functions, hormones, and characteristic molecular markers within each zone. This figure highlights the functional zonation of the adrenal gland in which aldosterone biosynthesis (*CYP11B2*) is predominantly localised to the zona glomerulosa, cortisol and androgen production to the deeper cortical layers, progenitor-associated markers (*GLI1*, *RSPO3*) to the subcapsular niche, and catecholamine synthesis to the neural crest-derived medulla. Created in BioRender. Zaidi, N. (2026) https://BioRender.com/lweke8s.

The adrenal cortex is organised into three concentric zones whose distinct steroidogenic identities are determined by zone-restricted enzyme expression rather than substrate availability [64,65]. The outermost ZG, situated immediately beneath the fibrous capsule (Figure 1), is the exclusive anatomical locus of mineralocorticoid biosynthesis. Its functional exclusivity is conferred at the molecular level by zone-restricted expression of *CYP11B2*, the aldosterone synthase enzyme that catalyses the three terminal steps of aldosterone biosynthesis and is entirely absent from the deeper cortical zones [66,67]. The ZF and ZR diverge from this programme at the level of *CYP17A1* expression, directing steroidogenesis toward cortisol and adrenal androgens respectively [68,69,70].

**Figure 2:**
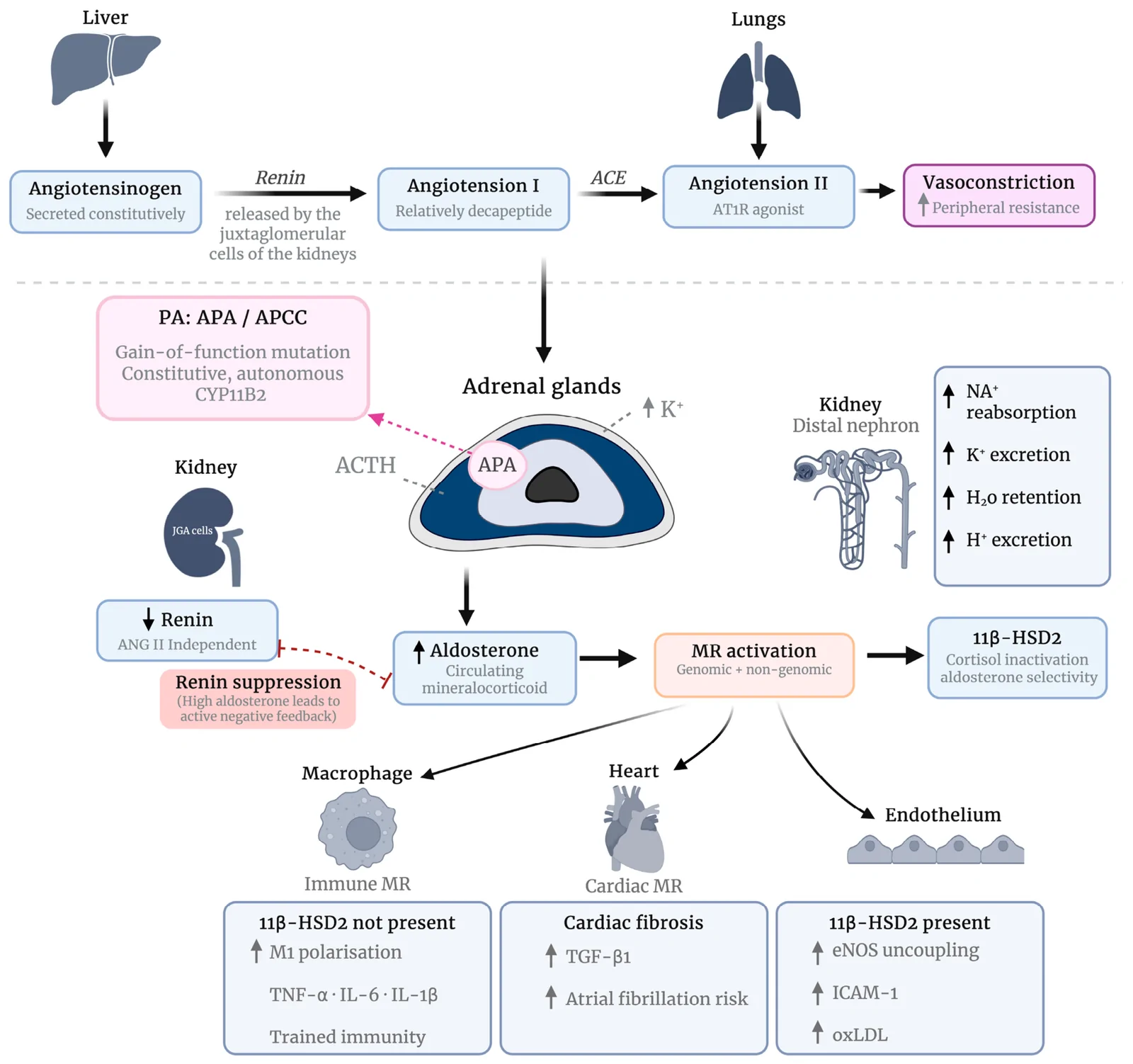
Uncoupling of the Renin–Angiotensin–Aldosterone System and tissue-specific consequences of autonomous MR activation in PA. The upper panel depicts physiological RAAS signalling, in which renin-driven angiotensin II generation stimulates aldosterone secretion under negative-feedback governance [43,44,56]. The lower panel illustrates pathological uncoupling in PA, where APAs harbouring gain-of-function somatic mutations drive constitutive *CYP11B2* expression and renin-independent aldosterone excess [45,46,47,49]. Sustained MR activation produces tissue-specific effects across multiple compartments, including sodium retention and potassium excretion within the 11β-HSD2-protected distal nephron [31,81,82]; pro-inflammatory macrophage polarisation and trained immunity-like responses in immune-lineage cells that lack substantial 11β-HSD2 activity [83,84,85]; the 11β-HSD2 tissue selectivity principle is established elsewhere [31,83,84,85]; cardiac fibrosis and arrhythmogenic remodelling [83,86,87]; and eNOS uncoupling with endothelial pro-adhesive activation [88,89,90]. Created in BioRender. Zaidi, N. (2026) https://BioRender.com/rgrbu1h.

*CYP11B2* transcription is governed by two convergent afferent signals within the intact RAAS: angiotensin II (AngII) acting at the Gq-coupled AT1 receptor, and extracellular potassium concentration, both engaging the same obligate intracellular effector (Figure 2) [43,44,56]. AT1R activation drives phospholipase C-dependent inositol trisphosphate generation and endoplasmic reticulum calcium release; potassium elevation directly depolarises the zona glomerulosa plasma membrane, facilitating calcium influx through voltage-gated calcium channels [56,71,72,73,74]. These mechanistically distinct stimuli converge upon cytosolic calcium accumulation, which activates calcium/calmodulin-dependent protein kinase I (CaMKI), the predominant CaMK isoform regulating *CYP11B2* transcription. This has been confirmed in human adrenocortical reporter assays in which constitutively active CaMKI, but not CaMKII, stimulated *CYP11B2* promoter activity and drove transcriptional upregulation of the NGFI-B family nuclear receptors NURR1 and NOR1 [75,76,77,78].

The resulting transcriptional response is biphasic and intrinsically self-limiting. The acute phase is governed by StAR-mediated cholesterol transfer and *CYP11A1*-mediated pregnenolone synthesis [67]; the delayed phase, beginning within one to two hours, is governed by CaMKI-dependent *CYP11B2* transcription. Self-limitation is actively enforced at both levels: AngII simultaneously induces the RNA-binding proteins *BTG2* and *ZFP36L2*, which promote mRNA decay of the same pro-steroidogenic transcripts they co-induce, constituting a mixed incoherent feedforward loop that curtails *CYP11B2* mRNA accumulation and ensures resolution of each steroidogenic episode within hours of stimulus withdrawal [79,80]. Rising aldosterone suppresses renin secretion, withdrawing the AngII stimulus and extinguishing *CYP11B2* transcription—a self-terminating negative-feedback circuit whose cell-autonomous disruption by somatic mutation constitutes the molecular origin of PA.

### 3.2. Somatic Driver Mutations: A Convergent Calcium Channelopathy

The mechanistic basis for autonomous mineralocorticoid hypersecretion has been substantially, albeit incompletely, elucidated over the past fifteen years through advances in next-generation sequencing (NGS) and *CYP11B2* immunohistochemistry-guided tumour sampling [68,69,91]. Four canonical somatic driver genes have been identified, *KCNJ5*, *ATP1A1*, *ATP2B3*, and *CACNA1D*, whose structurally distinct mutations converge upon a single pathological output: constitutive intracellular calcium elevation and renin-independent *CYP11B2* transcription [46,47,49,59,92]. The frequency and clinical correlates of each mutation differ substantially across APA subtypes, and the immunological consequences of these distinct calcium signalling dysregulations have not yet been systematically compared.

#### 3.2.1. KCNJ5 Mutations

The genomic taxonomy of APAs was established in 2011 when Choi and colleagues identified two recurrent somatic missense substitutions in *KCNJ5* at residues G151R and L168R by whole-exome sequencing of 22 surgically resected adenomas [45]. *KCNJ5* encodes Kir3.4, an inward rectifier potassium channel whose selectivity filter is disrupted by both substitutions, permitting aberrant sodium influx, constitutive membrane depolarisation, and secondary calcium entry through voltage-gated channels that drives autonomous *CYP11B2* transcription [45,93,94]. *KCNJ5* mutations are the most prevalent somatic driver identified to date, accounting for 40 to 65% of surgically resected APAs across international cohorts, though with a markedly higher prevalence in East Asian female patients than in European male patients [45,93,94]. This demographic and geographic asymmetry carries a direct methodological implication for the immunological framework developed in this review: mechanistic findings derived predominantly from *KCNJ5*-mutant cohorts, which constitute the majority of the surgical APA literature, may not be representative of PA across its full mutational spectrum, and their extrapolation to non-*KCNJ5* disease or to bilateral adrenal hyperplasia requires explicit caution.

#### 3.2.2. ATP1A1 and ATP2B3 Mutations

Loss-of-function mutations in *ATP1A1*, encoding the alpha-1 subunit of the Na^+^/K^+^-ATPase, and *ATP2B3*, encoding the plasma membrane Ca^2+^-ATPase type 3, were identified by Beuschlein and colleagues through massively parallel sequencing of APAs lacking *KCNJ5* mutations [49]. Though mechanistically distinct, both converge upon constitutive intracellular calcium elevation. *ATP1A1* mutations abolish sodium extrusion, generating pathological inward Na^+^ and H^+^ leakage that depolarises the membrane through a mechanism functionally convergent with *KCNJ5* gain-of-function [61,63]. *ATP2B3* mutations impair cytosolic calcium clearance, sustaining pathologically elevated baseline calcium independently of membrane potential [46,49]. Together, these mutation classes account for approximately 10 to 15% of surgically resected adenomas and are enriched in male patients and smaller tumours relative to *KCNJ5*-mutant disease [46,49].

#### 3.2.3. CACNA1D Mutations

Gain-of-function mutations in *CACNA1D*, encoding the Cav1.3 L-type voltage-gated calcium channel, were identified independently by Scholl and colleagues and by Azizan and colleagues in 2013 as a third mechanistically distinct pathway to constitutive ZG activation [46,47]. Unlike *KCNJ5* and *ATP1A1* mutations, which depolarise the plasma membrane to secondarily recruit voltage-gated calcium channels, *CACNA1D* mutations directly lower the channel opening threshold, producing constitutive calcium influx at resting membrane potentials without requiring an upstream depolarising event [46,47]. Accounting for approximately 9% of surgically resected APAs, *CACNA1D* mutations are distributed more evenly across sexes than *KCNJ5*-mutant disease and are represented in both Asian and European cohorts [46,47]. Germline *CACNA1D* mutations additionally underlie PASNA syndrome (PA, seizures, and neurological abnormalities), establishing *CACNA1D* as a channel whose gain-of-function consequences extend beyond adrenal steroidogenesis [46].

#### 3.2.4. Emerging Driver Mutations

Beyond these four canonical drivers, somatic mutations in *CACNA1H*, *CLCN2*, *CTNNB1*, *GNA11*, and *GNAQ* have been identified in emerging sequencing datasets, confirming that the somatic landscape of PA remains incompletely characterised [95,96,97,98,99,100,101,102]. Notably, *CTNNB1* mutations activating the Wnt/β-catenin pathway are enriched in APAs arising in specific hormonal contexts, including pregnancy and puberty, and represent a mechanistically distinct driver whose steroidogenic and potentially inflammatory consequences differ from calcium channelopathy-driven subtypes [97,98,100]. A substantial proportion of APAs, particularly those arising in male patients and in bilateral adrenal disease, remain without an identified somatic driver mutation, underscoring the incompleteness of the current genomic taxonomy.

All variants in the canonical driver genes converge towards an increase in intracellular calcium concentration and activation of calcium-dependent signalling, which plays a central role in CYP11B2 upregulation [43,44,45,46,47,48,49,56,103,104,105,106] The sustained calcium signal drives constitutive activation of CaMKI-dependent transcription factor inputs at the *CYP11B2* promoter, ensuring that constitutive *CYP11B2* transcription cannot be extinguished by disruption of any single upstream regulator. *CYP11B2* encodes aldosterone synthase, the rate-limiting mitochondrial enzyme catalysing the three terminal steps of mineralocorticoid biosynthesis: 11-beta-hydroxylation of 11-deoxycorticosterone to corticosterone, 18-hydroxylation to 18-hydroxycorticosterone, and 18-oxidation to aldosterone [104,105,106,107]. The chronic calcium signal generated by somatic mutations subverts the temporal self-limitation of this process, enforcing constitutive, high-level *CYP11B2* transcription irrespective of RAAS activity, dietary sodium intake, or extracellular potassium flux [56].

## 4. The Aldosterone–Immune Axis: Mineralocorticoid Receptor Signalling

The mechanistic bridge from autonomous adrenal secretion to systemic inflammatory disease is the MR. Renin-independent aldosterone hypersecretion, a convergent pathophysiological output of APA somatic mutations regardless of their genomic origin [45,108], is delivered without negative-feedback termination to a receptor whose expression is not confined to the renal tubular epithelium [109,110]. Transcriptomic and immunohistochemical evidence confirms MR expression across innate and adaptive immune lineages [32,33], macrophages [110], dendritic cells [111], and neutrophils [112]; MR expression has also been demonstrated in cardiomyocytes [34,35]; endothelial cells [36,113]; VSMCs [36]; adipocytes of both brown and white depots [37,38,114]; and hypothalamic and hippocampal neurones [39,40,115]. This anatomical breadth maps the inflammatory and structural consequences of autonomous aldosterone secretion onto the major organ system implicated in PA morbidity.

### 4.1. Genomic and Non-Genomic MR Signalling: Tissue Context and Ligand Selectivity

The biological consequences of MR activation are determined not by receptor occupancy alone but by the enzymatic steroid-metabolising environment of the target cell, the conformational state induced by the activating ligand, and the coregulator repertoire available for transcriptional complex assembly [30,116]. Beyond this classical genomic programme, MR activation initiates rapid non-genomic signalling cascades occurring within minutes of ligand binding, independently of de novo transcription or translation [117]. The MR has two primary physiological agonist ligands in humans, aldosterone and cortisol, while progesterone functions as an endogenous competitive antagonist [118]. Although the canonical role of aldosterone is the regulation of electrolyte flux across transporting epithelia, MR expression extends well beyond this compartment, encompassing immune-lineage cells, cardiomyocytes, endothelial cells, VSMCs, and central neurones [30,119].

In classical aldosterone-sensitive epithelia, including the renal cortical collecting duct, distal colonic mucosa, and salivary gland ducts, locally abundant 11β-HSD2 expression enforces this selectivity by rapidly converting active cortisol into cortisone [29,120]. This *HSD11B2*-mediated protective mechanism also operates in specific vascular beds [121] and discrete subpopulations of hypothalamic neurons [122]. Conversely, the MR in cardiomyocytes, monocyte/macrophages, adipocytes, and hippocampal neurons lacks this protection and is predominantly occupied by glucocorticoids under basal physiological conditions [123,124,125]. Once bound to the receptor, these agonists are functionally non-equivalent due to distinct ligand-induced conformations within the ligand-binding domain. MR/GR LBD chimeras demonstrate that receptor binding and activation are not equivalent for cortisol, whereas aldosterone promotes receptor N/C-domain interaction and recruits ligand-selective coactivators [118].

At baseline, the unliganded cytoplasmic MR is maintained in a transcriptionally inactive, high-affinity ligand-binding conformation by a multiprotein chaperone complex comprising HSP90, HSP70, and the immunophilins FKBP51 and FKBP52 [126,127]. Aldosterone binding displaces this complex, initiating nuclear translocation, receptor dimerisation, and assembly of transcriptional coregulator complexes at hormone-response element loci [128,129], followed by early SGK1-dependent NEDD4-2 phosphorylation and ENaC stabilisation and later synthesis of ENaC subunits and Na+/K+-ATPase components [81,82]. In living cultured cells, aldosterone-bound MR translocates to the nucleus within approximately 10–20 min, although the kinetics and transcriptional consequences of MR activation vary across cell types and experimental conditions [128]. Although aldosterone and cortisol bind the MR with comparable affinity, they induce structurally distinct receptor conformations [130,131]. Glucocorticoid receptor (GR) co-expression can further shape MR transcriptional activity in a locus- and ligand-dependent manner, with GR contributing to productive MR/aldosterone chromatin engagement [132].

Parallel to the nuclear genomic programme, ligand-bound MR initiates rapid signalling cascades independently of *de novo* transcription or translation, with ERK1/2 phosphorylation, PKC activation, and IP3-mediated calcium mobilisation detectable within minutes of aldosterone exposure [117,133,134,135,136,137]. In murine experimental models, these cascades have been associated with membrane-associated MR anchored within caveolar microdomains through scaffolding protein interactions [138], and with crosstalk involving the G-protein-coupled oestrogen receptor GPER and Src kinase-dependent EGFR transactivation [88,134,139]. In experimental vascular and cardiac systems, non-genomic and genomic signalling can operate simultaneously, with non-genomic kinase activation augmenting genomic transcriptional responses [109,140]. Whether equivalent crosstalk occurs in immune-lineage cells has not been directly demonstrated. In PA, sustained renin-independent aldosterone excess may prolong engagement of these signalling pathways, potentially increasing the duration of downstream inflammatory signalling in tissues lacking 11β-HSD2 protection [141,142].

The transcriptional output of aldosterone-bound MR in immune-lineage cells is predominantly pro-inflammatory in experimental systems, diverging from the broadly anti-inflammatory programme of the GR, which suppresses inflammatory gene expression through direct trans-repression of NF-κB and AP-1 [143,144]. In monocytes and macrophages, aldosterone-bound MR engages AP-1 at inflammatory gene promoters through a Rac1 GTPase-dependent mechanism [145], driving transcription of pro-inflammatory cytokines and chemokines [83], priming the NLRP3 inflammasome [146], and sustaining macrophage polarisation towards an M1-associated transcriptional state [147]. NADPH oxidase-dependent ROS generation has been demonstrated downstream of aldosterone-MR activation in vascular experimental systems [88,148], although direct confirmation in primary human monocyte-derived macrophages has not been established.

**Figure 3:**
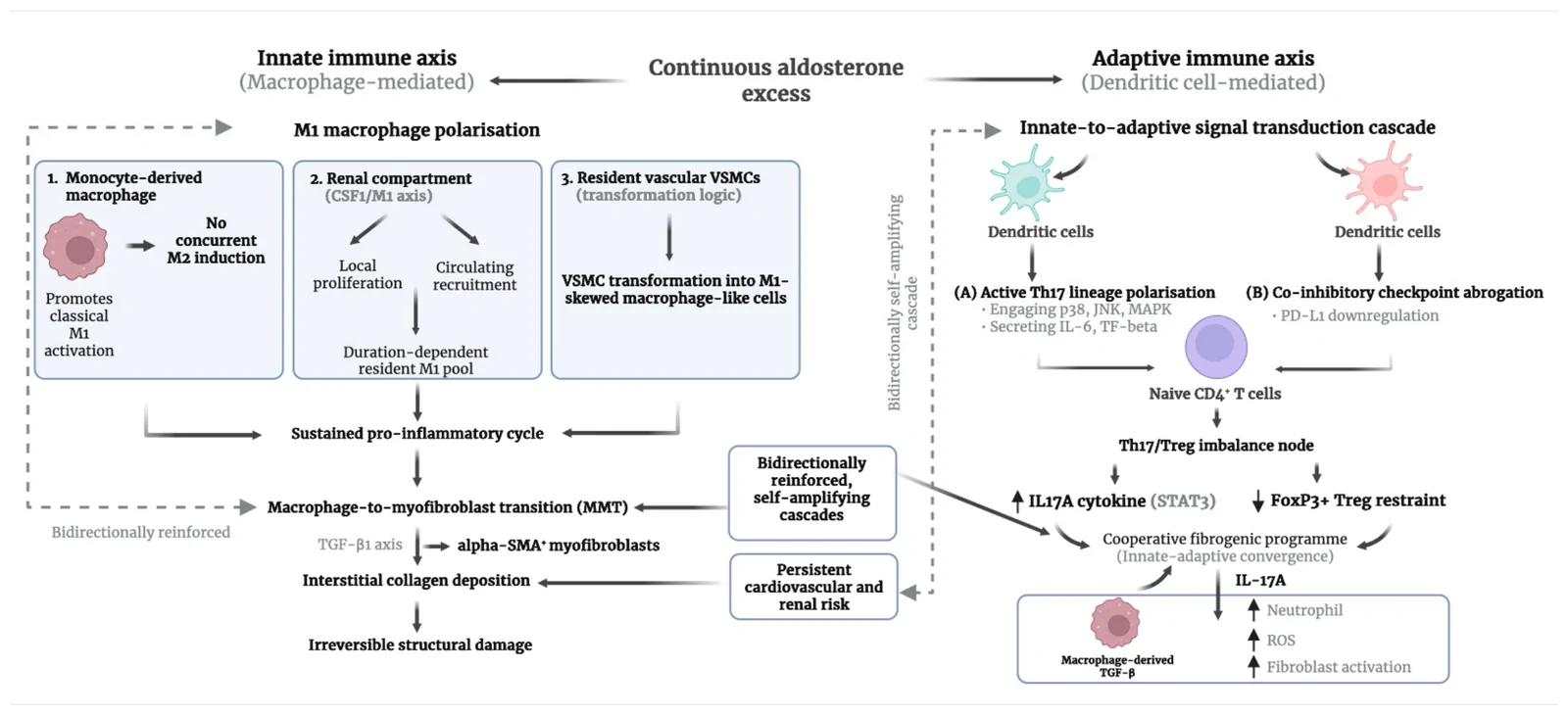
Model of aldosterone-driven immune programmes in primary aldosteronism (PA) and their fibrotic consequences, synthesised largely from rodent aldosterone-excess models with supporting human PA data. Aldosterone promoted renal macrophage proliferation via an MR/CSF1-dependent pathway, macrophage-to-myofibroblast transition via MR/TGF-β1 signalling, and transformation of vascular smooth muscle cells into macrophage-like cells [86,150,151]. In parallel, aldosterone enhanced Th17-mediated immunity, and Th17 expansion with relative Treg suppression was demonstrated in mineralocorticoid-excess models and in a cohort of PA patients, with Treg populations shown to protect against mineralocorticoid- and angiotensin II-induced vascular and cardiac injury [54,86,111,150,151,152,153,154]. These innate and adaptive programmes are proposed to converge on a shared TGF-β- and IL-17A-associated fibrogenic circuit, offering an integrative model for PA-associated target-organ damage. Created in BioRender. Zaidi, N. (2026) https://BioRender.com/k17u72x.

The continuous autonomous aldosterone excess of PA engages immune-lineage MR through two converging mechanisms that may become particularly relevant under conditions of autonomous secretion. First, the absence of 11β-HSD2 in monocytes, macrophages, and lymphocytes permits greater exposure of MR to aldosterone [31,43,147]. Second, aldosterone induces a ligand-selective MR conformation characterised by stronger NTD-LBD interdomain interaction than cortisol at equivalent receptor occupancy [130,149], while GR co-expression can further influence MR transcriptional activity in a locus- and ligand-dependent manner [132]. Together, these mechanisms provide a potential basis for sustained pro-inflammatory MR signalling during autonomous aldosterone secretion (Figure 3). The concentration-dependence and reversibility of this response in human PA immune-lineage cells have not been directly characterised.

### 4.2. Innate Immune Activation and Macrophage Polarisation

The disproportionate organ damage of PA requires a cellular explanation that extends beyond haemodynamic mechanisms. The most direct human evidence implicating aldosterone in immune cell reprogramming was provided by van der Heijden and colleagues in a prospective cohort of patients with biochemically confirmed PA matched to blood-pressure-equivalent essential hypertension controls: PA was associated with a significantly elevated monocyte-to-lymphocyte ratio, a transcriptomically reprogrammed monocyte pool, and upregulated TNF-α in monocyte-derived macrophages, each effect operating independently of conventional circulating inflammatory markers including hsCRP, IL-6, and IL-18 [52]. This monocyte reprogramming was shown to produce a directional M1 macrophage phenotype, as confirmed *in vitro.* In primary human monocyte-derived macrophages from healthy donors exposed to exogenous aldosterone, aldosterone promoted classical M1 activation characterised by upregulation of iNOS and ROS production. Eplerenone, a selective MR antagonist, redirected primary human monocyte-derived macrophages from a classically activated phenotype towards an alternative activation state, accompanied by changes in macrophage activation markers [84].

Conditional deletion of MR from macrophages in mice exposed to DOCA/salt hypertension attenuated blood pressure elevation and protected against cardiac fibrosis despite continued macrophage recruitment, supporting a direct role for macrophage MR signalling in the development of structural injury [147]. In experimental aldosterone-salt models, aldosterone exposure is associated with macrophage activation, hypertension, cardiac and renal structural injury, and fibrosis, while MR antagonism attenuates these responses [155]. Macrophage depletion with liposomal clodronate reduced macrophage infiltration and inflammatory/profibrotic responses but did not fully prevent established fibrosis, suggesting that established structural remodelling may become progressively less dependent on continued macrophage accumulation [155]. In PA, the duration of autonomous aldosterone excess before treatment may therefore directly determine the proportion of organ damage that is reversible, consistent with the clinical observation that earlier treatment is associated with greater structural regression [22,156] (Figure 3).

This M1 dominance extends beyond circulating monocyte-derived macrophages. *In vitro* experiments using murine VSMCs showed that aldosterone acting through VSMC MR promoted phenotypic transformation towards macrophage-like cells through MR/M-CSF/M-CSFR signalling, with an approximately 5.5:1 M1-skewed phenotype confirmed by flow cytometry [150]. A comparator experiment in which TGF-β induced VSMC transformation produced a more M2-associated phenotype, supporting the specificity of the observed M1 directionality for aldosterone-MR signalling [150]. Whether this VSMC-to-macrophage transformation operates in human PA vascular tissue remains unconfirmed; it has not been demonstrated in spatially resolved human PA tissue studies and should be interpreted as a mechanistic hypothesis derived from murine and *in vitro* data. In the renal compartment, single-cell RNA sequencing in a preclinical aldosterone-infusion murine model demonstrated MR-dependent expansion of renal macrophage populations, with an M1-associated phenotype predominating across the identified subpopulations [151]. Esaxerenone—a non-steroidal, highly selective MR antagonist approved in Japan for hypertension with renal complications—substantially attenuated this proliferative expansion, confirming MR dependence pharmacologically [151]. Through a parallel pathway, nuclear MR accumulation in macrophages upregulates TGF-β1 and induces macrophage-to-myofibroblast transition, generating alpha-SMA-positive myofibroblasts as the proximate source of interstitial collagen deposition in preclinical models, blocked by esaxerenone through antagonism of MR nuclear accumulation [86]. Both findings derive from preclinical murine aldosterone infusion models and require confirmation in human PA renal tissue.

In the mechanistic sequence from aldosterone excess to structural organ injury, macrophage activation represents an important upstream component at which MR antagonism may interrupt fibrogenic progression before structural remodelling becomes established [25,147]. The duration-dependence of this potential window in human PA has not been prospectively defined; however, the greater structural regression observed following earlier treatment is consistent with the hypothesis that reversibility may decline as structural remodelling progresses beyond the initiating aldosterone stimulus [25].

### 4.3. Adaptive Immune Crosstalk: Th17 Expansion and Treg Suppression

The excess organ damage in PA is not solely the product of macrophage-mediated innate immune injury. In a human clinical study, Imiela and colleagues demonstrated a significant Th17/Treg imbalance in patients with PA that was not replicated in blood-pressure-matched patients with resistant hypertension, providing direct human evidence that adaptive immune dysregulation is an intrinsic feature of aldosterone excess rather than a consequence of elevated blood pressure [54]. This human observation demands a mechanistic explanation; the preclinical framework that provides it operates through a dendritic cell-mediated innate-to-adaptive signal transduction cascade. The innate immune dysregulation established by aldosterone-MR signalling propagates into the adaptive compartment through MR-dependent activation of dendritic cells, which, in experimental murine models, serve as the cellular conduit between innate immune priming and Th17 polarisation [111] (Figure 3).

In experimental murine models, aldosterone-mediated MR activation in dendritic cells engaged p38 and JNK MAPK pathways and drove IL-6 and TGF-β secretion, polarising naive CD4-positive T cells toward the Th17 lineage [111]. Antigen-presenting cell-independent assays confirmed that dendritic cell intermediation is required; in the complete absence of APCs, no T cell modulation was observed, establishing that direct T cell MR ligation is not an operative Th17 polarisation signal [111]. Concurrent aldosterone-mediated downregulation of PD-L1 on dendritic cells abrogated the co-inhibitory checkpoint governing T cell activation threshold, such that Th17 induction and checkpoint suppression operate as mechanistically distinct but cooperative consequences of the same upstream MR activation event [111]. Conditional genetic confirmation that dendritic cell MR is causally necessary was provided by Wang and colleagues, who demonstrated that dendritic cell-specific MR deletion prevented aldosterone-induced Th17 differentiation and attenuated blood pressure elevation in a murine model [157].

In the DOCA-salt murine model, mineralocorticoid excess drove simultaneous Th17 activation and FoxP3^+^ Treg downregulation; spironolactone, a first-generation steroidal MR antagonist with cross-reactivity at androgen and progesterone receptors, prevented both responses, establishing MR dependence pharmacologically [152]. Anti-IL-17A antibody treatment reduced profibrotic mediators and collagen deposition in the heart and kidney independently of blood pressure reduction, establishing IL-17A as an independent fibrogenic effector acting through STAT3 to amplify endothelial oxidative stress, neutrophil recruitment, and fibroblast activation [152]. Treg adoptive transfer to aldosterone-infused mice prevented VCAM-1 upregulation, macrophage and T cell infiltration of the aorta and renal cortex, NADPH oxidase activation, and resistance artery remodelling, while exerting a more modest effect on blood pressure than on structural endpoints [153]. In the AngII murine model, Treg transfer prevented cardiac fibrosis and hypertrophy entirely without significant haemodynamic effect [154]. This dissociation between structural protection and blood pressure reduction in experimental models suggests that the Th17/Treg axis may operate as a structural remodelling effector whose consequences extend beyond what blood pressure monitoring captures. The mechanistic Th17/Treg cascade described above derives entirely from experimental murine mineralocorticoid excess models. Whether an equivalent cascade operates in human PA through the same stepwise mechanism requires confirmation by prospective human tissue studies and circulating immune phenotyping with genotype-stratified cohorts. The human Th17/Treg imbalance documented by Imiela and colleagues [54] confirms that the net imbalance is operationally present in the clinical PA population but does not characterise the individual mechanistic steps.

What emerges from the convergent mechanistic and human clinical evidence is not a linear inflammatory sequence but a self-amplifying, bidirectionally reinforced cascade without an intrinsic resolution mechanism under continuous mineralocorticoid excess. Aldosterone simultaneously enforces macrophage M1 polarisation through direct MR activation, imposes Th17 skewing upon naive T cells through dendritic cell intermediation, suppresses the FoxP3^+^ Treg population that would otherwise provide counter-regulatory restraint [151,152,153], and recruits additional M1-polarised macrophage-like effectors at the vessel wall through VSMC transformation in experimental models [150]. Each of these effector pathways is sustained without feedback attenuation for the duration of autonomous aldosterone excess, generating a duration-dependent, partially irreversible innate and adaptive immune dysregulation that constitutes the cellular substrate for the systemic inflammatory state and the pressure-independent target-organ damage that define the clinical phenotype of PA [22,25,54,158].

## 5. Systemic Inflammation in Primary Aldosteronism: Propagation of the Aldosterone–Immune Axis

The innate and adaptive immune programmes characterised above propagate systemically through four principal mechanisms. MR-mediated endothelial transformation across the systemic arterial tree generates a sustained pro-adhesive and pro-oxidant vascular surface through NADPH oxidase activation and eNOS uncoupling [157,158]. Bone marrow activation expands myeloid progenitors and continuously replenishes the circulating monocyte pool with cells carrying an aldosterone-primed transcriptional bias [52,159]. Monocyte-macrophage lineage cells may acquire a trained innate immunity phenotype through MR-dependent fatty acid synthesis and H3K4me3 epigenetic reprogramming at inflammatory gene promoters [85]. Central MR activation may amplify sympathetic outflow through neuroendocrine pathways partly independent of peripheral haemodynamic load [160]. Critically, none of these mechanisms generates the systemic cytokine spillover required to activate conventional acute-phase responses: hsCRP and leucocyte counts are typically comparable between PA patients and blood-pressure-matched essential hypertension controls [52,159], reflecting a disease in which inflammatory and fibrotic signalling is predominantly confined to tissue microenvironments. This tissue-confined architecture is precisely why conventional circulating biomarker panels underestimate the inflammatory burden of PA and why structural organ damage accumulates despite apparently unremarkable systemic inflammatory indices.

### 5.1. Aldosterone-Specific Vascular Inflammation as the Circulating Hallmark

In PA, continuous autonomous aldosterone excess occupies endothelial MR without the pulsatile interruption and feedback-governed concentration ceiling that limit receptor occupancy under physiological renin–angiotensin governance [43,44,56]. Endothelial MR activation initiates rapid Src kinase-dependent transactivation of the epidermal growth factor receptor, coupling through ERK1/2 phosphorylation to NADPH oxidase activation and superoxide generation within minutes of aldosterone exposure, independently of transcriptional regulation [143]. NADPH oxidase-derived superoxide reacts with nitric oxide (NO) to form peroxynitrite, depleting the NO pool and oxidising the essential eNOS cofactor tetrahydrobiopterin [89,161]. Tetrahydrobiopterin oxidation uncouples eNOS from its L-arginine substrate, diverting the enzyme from NO synthesis to further superoxide production, establishing a partially self-sustaining oxidative cycle [89]. The clinical consequence of this endothelial dysfunction is detectable in human PA. Impaired endothelium-dependent flow-mediated vasodilation has been demonstrated in patients with confirmed PA relative to blood-pressure-matched essential hypertension controls, with partial rather than complete restoration following MR antagonist administration [162], consistent with a component of the eNOS uncoupling cycle persisting after reduction of MR stimulation (Table 1).

Surgical adrenalectomy, by eliminating the autonomous aldosterone source, normalises long-term AF risk toward essential hypertension-comparable levels [163]. In contrast, medically treated PA patients in whom MR antagonist therapy fails to restore renin activity retain significantly elevated AF risk despite blood pressure control [164], supporting persistent MR-dependent signalling as an important contributor to residual cardiovascular risk. AF risk remains transiently elevated during the first three years following treatment initiation [87], which may reflect the time required for structural and functional cardiovascular remodelling following elimination of the autonomous aldosterone signal.

**Table 1:** Mechanistic evidence for the systemic inflammation in primary aldosteronism.

Mechanism	Model	Primary Findings
**1. Vascular inflammation**		
NADPH oxidase activation	In vivo (rat, DOCA-salt mineralocorticoid model)	Mineralocorticoid excess upregulates vascular NADPH oxidase; enhanced O_2_^−^ production [165]
Impaired flow-mediated vasodilation	Human cohort, PA vs. essential hypertension	MR antagonism partially restored endothelium-dependent vasodilation in PA [162]
Aldosterone-driven AF	Human cohort, adrenalectomy vs. medical therapy	Adrenalectomy normalised AF risk; unsuppressed renin retained elevated risk [163,164]
**2. Oxidative stress, endothelial activation and haemostasis**		
Systemic oxidative lipid burden	Human cohort, PA vs. essential hypertension	NADPH oxidase activity and urinary isoprostanes fell after adrenalectomy [166]
Endothelial–thrombotic marker elevation	Human cohort, PA vs. essential hypertension	Oxidised LDL, ICAM-1, and von Willebrand factor were elevated [167,168]
Macrophage-driven atherosclerotic plaque	In vivo, ApoE-knockout mice	Aldosterone accelerated atherosclerotic lesion development via macrophage oxidase [169]
ICAM-1-dependent atherogenesis	In vivo, ApoE/ICAM-1 double-knockout mice	ICAM-1 deletion abolished aldosterone-induced plaque formation [170]
Vascular COX-2/MCP-1 induction	In vivo, mineralocorticoid excess rodent model	Eplerenone attenuated inflammatory mediator upregulation in vascular tissue [171]
PAI-1-mediated renal injury	In vivo, PAI-1-deficient mice	PAI-1 deficiency attenuated aldosterone-induced glomerular injury independently of pressure [172]
**3. Bone marrow niche activation**		
Bone marrow myeloid activation	Human cohort, PET-CT imaging	Bone marrow 18F-FDG uptake and monocyte–lymphocyte ratio were elevated [52]
Myeloid-to-lymphoid compositional shift	Human cohort, 346 propensity-matched patients	Neutrophil–lymphocyte and monocyte–lymphocyte ratios rose with aldosterone concentration [159]
Trained myelopoiesis propagation	In vivo, murine beta-glucan model	Bone marrow transplantation transferred trained phenotype to naive recipients [173]
**4. Trained innate immunity**		
Fatty acid-driven trained immunity	*In vitro*, human monocyte-derived macrophages	MR-dependent H3K4me3 enrichment augmented cytokine and ROS responses [85]
Trained macrophage hyperresponsiveness	Human cohort, PA patient-derived macrophages	Oxidised LDL stimulation elicited augmented TNF-α versus hypertension controls [85]
Serum-transferable trained phenotype	Human cohort, healthy monocytes in PA serum	PA serum augmented TNF-α and IL-6 upon TLR stimulation [85]
Persistent trained hyperresponsiveness	Human cohort, phaeochromocytoma comparator	Catecholamine-induced H3K4me3 enrichment persisted four weeks post-cure [174]
**5. Sympathetic amplification**		
Central MR sympathetic amplification	Rodent model, nucleus tractus solitarius	11β-HSD2-expressing neurons conferred aldosterone selectivity at central MR [122]
Reversible sympathetic overactivity	Human cohort, microneurography	Muscle sympathetic nerve activity fell significantly after adrenalectomy [175]
Aldosterone-independent autonomic dysregulation	Human cohort, APA vs. essential hypertension	Heart rhythm complexity correlated with aldosterone–renin ratio, improved post-adrenalectomy [176]

### 5.2. Oxidative Stress, Endothelial Activation, and Haemostatic Perturbation

Autonomous mineralocorticoid excess imposes three converging pathological processes upon the systemic vasculature. NADPH oxidase-driven oxidative stress generates a systemic lipid oxidative burden detectable above that observed in blood-pressure-matched essential hypertension [166]. NF-kB-dependent endothelial activation promotes leukocyte recruitment across the vascular endothelium [88]. MR-dependent plasminogen activator inhibitor-1 induction impairs fibrinolytic capacity, contributing to a prothrombotic vascular phenotype [177,178]. These processes are mechanistically linked rather than independent: oxidative stress promotes endothelial activation, endothelial activation sustains macrophage recruitment, and haemostatic impairment amplifies thrombotic susceptibility [178].

Patients with PA demonstrate significantly elevated NADPH oxidase activity and urinary isoprostane concentrations relative to blood-pressure-matched essential hypertension controls, with both markers decreasing significantly after adrenalectomy in unilateral disease, establishing that this oxidative burden is driven by autonomous aldosterone excess rather than haemodynamic load [166]. This oxidative burden is sufficient in principle to drive oxidative modification of circulating LDL. Circulating oxidised LDL is also elevated in PA relative to matched essential hypertension controls alongside ICAM-1 and von Willebrand factor [167,168]. However, direct causal linkage between aldosterone excess and foam-cell biology in human PA plaque tissue remains inferential and requires further translational investigation. In ApoE knockout mice, aldosterone administration independently drives macrophage NADPH oxidase activation and accelerates atherosclerotic lesion development [169], and the causal necessity of ICAM-1 for aldosterone-driven atherogenesis has been established in ApoE/ICAM-1 double knockout mice protected from aldosterone-induced plaque [170]. Direct mechanistic demonstration of the complete foam cell propagation axis in human PA plaque tissue has not been established and represents a translational priority.

Aldosterone-driven NF-κB-dependent transcriptional activation drives coordinated upregulation of COX-2 and MCP-1 in vascular tissue, with eplerenone attenuating these inflammatory mediators in mineralocorticoid excess rodent models [171]. Circulating ICAM-1 is significantly elevated in PA patients relative to individually matched essential hypertension controls, independently predicting microalbuminuria and left ventricular hypertrophy on multivariate regression alongside plasma aldosterone concentration [168]. MR-dependent induction of PAI-1 in vascular tissue impairs fibrinolytic capacity: PAI-1 deficiency attenuates aldosterone-driven glomerular injury independently of blood pressure in experimental models, suggesting, in experimental PAI-1-deficient models, that PAI-1 is a causally necessary effector of mineralocorticoid-mediated glomerular injury [172]. Whether circulating PAI-1 is elevated in human PA and normalises following adrenalectomy or MR antagonism requires verification in a dedicated human PA cohort [158,179].

### 5.3. Bone Marrow Niche Activation: The Systemic Myeloid Supply Chain

The arterial wall macrophage infiltration that constitutes the primary effector mechanism of aldosterone-driven vascular inflammation cannot be sustained by tissue-resident macrophage proliferation alone and requires continuous replenishment from circulating monocytes derived from the bone marrow haematopoietic niche [180,181]. Direct human imaging evidence for enhanced haematopoietic activity in PA was provided by van der Heijden and colleagues in a cross-sectional PET-CT cohort demonstrating significantly elevated bone marrow 18F-FDG uptake in PA patients relative to matched essential hypertension controls [52]. Peripheral blood analysis in the same cohort revealed an elevated monocyte-to-lymphocyte ratio, consistent with enhanced myeloid mobilisation [52]. This myeloid dominance pattern was independently corroborated at larger scale by Rao and colleagues in 346 propensity-score-matched PA versus essential hypertension patients: absolute lymphocyte count was significantly lower in PA, neutrophil-to-lymphocyte ratio was significantly higher, and monocyte-to-lymphocyte ratio was significantly higher, with the strength of these correlations increasing proportionately with rising plasma aldosterone concentration, establishing an aldosterone-dose-dependent myeloid-to-lymphoid compositional shift that cannot be attributed to blood pressure alone [159].

The mechanistic basis for haematopoietic progenitor engagement was established in a murine model: systemic beta-glucan administration induced sustained expansion of myeloid-lineage progenitors persisting to 28 days, associated with IL-1β and GM-CSF signalling, and bone marrow transplantation from trained donors transferred this phenotype to naive recipients, establishing that HSPC-level imprinting propagates trained immunity across successive monocyte generations [173]. Whether autonomous aldosterone excess in PA engages an analogous HSPC-level reprogramming programme has not been directly demonstrated and constitutes the critical unresolved mechanistic question linking the bone marrow niche and trained immunity evidence in this section.

### 5.4. Trained Immunity as Systemic Inflammatory Memory

Aldosterone induces a trained immunity phenotype in human monocytes through MR-dependent activation of fatty acid synthesis, establishing a mechanistically distinct pathway from the mTOR-dependent immunometabolic reprogramming that mediates canonical beta-glucan trained immunity [85]. *In vitro,* aldosterone-exposed human monocytes differentiated to macrophages demonstrate augmented pro-inflammatory cytokine production and ROS upon restimulation with TLR-2 and TLR-4 ligands; this effect is substantially attenuated by MR antagonism and blunted by pharmacological inhibition of fatty acid synthesis [85]. At the epigenetic level, aldosterone promotes H3K4me3 enrichment at the promoters of fatty acid synthesis genes including *ACACA*, *FASN*, and *ELOVL6* and at pro-inflammatory cytokine loci including IL-6 and TNF-α [85].

In the human PA cohort prospectively characterised by van der Heijden and colleagues, subject-specific monocyte-derived macrophages from PA patients stimulated with oxidised LDL expressed significantly greater TNF-α than macrophages from essential hypertension controls; and monocytes from healthy donors differentiated in pooled PA patient serum showed augmented TNF-α production on TLR-2 stimulation and augmented IL-6 production on both TLR-2 and TLR-4 stimulation compared with cells differentiated in essential hypertension control serum [85]. This trained phenotype manifests at the macrophage differentiation stage, not in resting circulating monocytes, and is therefore invisible to standard circulating inflammatory biomarker panels.

A critical distinction must be made explicit. Whether the aldosterone-driven trained immunity phenotype persists after biochemical correction of PA by adrenalectomy or adequate MR antagonism has not been directly demonstrated in human PA patients. This is an open experimental question. The closest available human evidence comes from a pheochromocytoma comparator model, in which catecholamine-induced monocyte inflammatory hyperresponsiveness, including in vivo H3K4me3 enrichment at inflammatory pathway gene promoters, persisted for four weeks following surgical cure [174]. However, pheochromocytoma represents a distinct hormonal excess state, and this finding cannot be directly extrapolated to PA.

This distinction carries mechanistic importance that is directly relevant to clinical practice. The trained phenotype is not detectable in the peripheral blood compartment but is expressed upon tissue-stage macrophage differentiation, consistent with the pro-inflammatory milieu of the arterial wall where the aldosterone–immune axis exerts its structural consequences [53,85]. Because the epigenetic programme is operative at the tissue level while remaining invisible to standard circulating inflammatory biomarker panels, the inflammatory burden of PA is systematically underestimated by conventional risk stratification approaches that rely on hsCRP and leucocyte counts, with direct implications for treatment monitoring and the design of post-cure surveillance strategies [173,182].

The cardiovascular and renal damage burden in PA does not fully resolve upon biochemical cure, and residual risk is proportionate to the duration of aldosterone excess before treatment [25]. One mechanistically plausible explanation for this persistence is that reprogrammed bone marrow progenitors, as demonstrated in haematopoietic trained immunity models [173], continuously generate epigenetically imprinted monocyte progeny whose inflammatory hyperresponsiveness outlasts the hormonal event that initiated it, decaying progressively as trained progenitors are replaced by untrained progeny over a biological rather than biochemical timeframe.

Three convergent clinical observations are compatible with, though not specific to, this model. Carotid intima-media thickness in PA undergoes only slow and partial regression over a six-year follow-up period despite successful surgical or medical treatment [183]. Incident AF risk remains significantly elevated for up to three years in treated PA overall [87]; normalising to essential hypertension-comparable levels only after adrenalectomy [163], and remaining elevated with inadequate MR blockade defined by persistent renin suppression [164]. Diagnostic delay, documented in more than one-third of patients in an international cohort, permits accumulation of structural damage that targeted treatment cannot fully reverse [17]. These observations are equally consistent with structural fibrotic persistence and other mechanisms, but if the trained haematopoietic progenitor model is operative in PA, the post-treatment risk window may additionally reflect an epigenetic inflammatory component that persists beyond biochemical remission. Whether its duration directly reflects the propagation interval of a trained progenitor pool has not been established in any study linking bone marrow epigenetic status to incident clinical events in PA and constitutes a high-priority mechanistic question with direct implications for the timing and modality of treatment [85,158].

### 5.5. Central Sympathetic Amplification: The Neuroendocrine Multiplier

The systemic propagation of the aldosterone–immune axis extends to the central nervous system as a neuroendocrine amplifier. The anatomical substrate for central MR activation by aldosterone is characterised in rodent models within the nucleus of the solitary tract, where 11β-HSD2-expressing neurons confer aldosterone selectivity at the MR and project to sympathoexcitatory brainstem nuclei [122]. Lipophilic aldosterone penetrates the blood–brain barrier to access these central autonomic regulatory centres [122].

Clinical evidence for aldosterone-driven autonomic dysregulation in human PA rests on three mutually reinforcing lines of observation. Direct muscle sympathetic nerve activity measurement by microneurography in 14 biochemically confirmed PA patients found MSNA significantly elevated relative to normotensive controls but statistically comparable to essential hypertension controls, with significant reduction following adrenalectomy [175]. Earlier studies reported the opposite pattern of suppressed MSNA in PA, likely reflecting the competing suppressive effect of aldosterone-mediated volume expansion on cardiopulmonary baroreflex tone [184,185]. Nonlinear heart rhythm complexity analysis provides convergent evidence independent of haemodynamic confounding. In a prospective study of 20 APA patients undergoing adrenalectomy versus 25 essential hypertension controls, multiscale entropy metrics were significantly impaired in APA patients relative to controls independently of blood pressure, correlated significantly with the aldosterone-to-renin ratio and renin activity but not with blood pressure, and improved one year after adrenalectomy [176].

The immunological significance of this central pathway rests on the established capacity of catecholamines to independently induce trained immunity through β-adrenoreceptor-dependent epigenetic reprogramming [174]. PA therefore potentially exposes circulating monocytes to two mechanistically independent trained immunity inducers operating through non-overlapping intracellular pathways: the aldosterone/MR/fatty acid synthesis axis and the catecholamine/β-AR/cAMP axis [85,174]. Whether the resultant epigenetic burden is additive, synergistic, or partially redundant has not been investigated in any PA cohort and constitutes a high-priority mechanistic gap with direct therapeutic relevance.

## 6. Hypertensive Target-Organ Damage in Primary Aldosteronism

The mechanistic pathways characterised in the preceding sections converge on a clinical reality documented in population-scale human cohorts: PA inflicts cardiovascular and renal structural injury substantially in excess of what blood pressure elevation alone can explain. A meta-analysis of 31 studies enrolling 3838 PA patients and 9284 blood-pressure-matched essential hypertension controls documented significantly higher odds of left ventricular hypertrophy, AF, heart failure, stroke, coronary artery disease, and new-onset diabetes mellitus in PA over a median follow-up of 8.8 years [25]. In a retrospective cohort of 602 medically treated PA patients matched to 41,853 essential hypertension controls at equivalent blood pressure, those with PA carried a two-fold higher risk of myocardial infarction, heart failure hospitalisation, and stroke alongside substantially higher rates of AF, new-onset diabetes, chronic kidney disease, and all-cause mortality [186].

### 6.1. Cardiac Structural Damage and Functional Remodelling

The treatment-response gradient for AF mirrors the cardiac structural data and has been characterised with granular temporal resolution. In a Korean nationwide cohort of 1418 PA patients matched 1:5 to 7090 essential hypertension controls, incident AF risk peaked at one year following treatment initiation and gradually declined but remained significantly elevated up to three years in overall treated PA (aHR 3.02; *p* < 0.05) [87]. Adrenalectomy normalises long-term AF risk to essential hypertension-comparable levels in a prospective cohort with median 11.8-year follow-up [163], whereas patients in whom MR antagonist therapy fails to restore renin activity retain significantly elevated AF risk (adjusted HR 2.55; 95% CI 1.75–3.71) despite equivalent blood pressure control [164]. The post-treatment three-year persistence of elevated AF risk even after adrenalectomy is consistent with the time required for reversal of established atrial fibrosis. This supports the interpretation that structural consequences of autonomous aldosterone excess outlast the hormonal event that initiated them. The structural claim rests on human cohort evidence; whether an additional epigenetic inflammatory component contributes to this persistence remains mechanistically plausible but has not been directly demonstrated in PA patients.

### 6.2. Renal Compartment Injury

Renal damage in PA is clinically detectable as microalbuminuria and reduced estimated glomerular filtration rate at frequencies exceeding those of blood-pressure-matched essential hypertension. The Monticone 2018 meta-analysis identified significantly higher rates of renal insufficiency and microalbuminuria in PA versus essential hypertension controls [25], while the Hundemer 2018 cohort documented significantly higher incident chronic kidney disease in medically treated PA versus matched essential hypertension despite similar blood pressure [186].

The renal injury programme operates through three converging MR-dependent pathways whose mechanistic basis is established in preclinical models with partial human data. First, aldosterone drives local M1 macrophage expansion in the renal interstitium via MR-dependent upregulation of colony-stimulating factor 1, generating duration-dependent accumulation of a tissue-resident M1 macrophage pool; single-cell RNA sequencing in preclinical aldosterone infusion models demonstrated MR-dependent expansion across all three renal macrophage subpopulations with M1 dominance in each [151]. Second, podocytes express functional MR but have limited 11β-HSD2 activity, leaving the glomerular filtration barrier relatively exposed to aldosterone in a manner analogous to immune-lineage cells [187]. Third, aldosterone promotes glomerular injury through MR-genomic PAI-1 induction, and PAI-1 deficiency attenuates aldosterone-driven glomerular injury independently of blood pressure in experimental models [172].

Targeted treatment of PA is followed by an acute eGFR decline attributable to elimination of aldosterone-driven glomerular hyperfiltration, unmasking the true extent of underlying structural nephron loss [188]. In a retrospective cohort of 134 PA patients, eGFR declined by a mean of 15.3 ± 14.2 mL/min following adrenalectomy or MR antagonist initiation [188]. This decline reflects haemodynamic correction of pathological glomerular hyperfiltration rather than treatment-induced damage; it should not prompt treatment discontinuation in the absence of other acute kidney injury indicators, and its magnitude is proportionate to the pre-treatment degree of aldosterone-driven hyperfiltration.

### 6.3. Cerebrovascular, Metabolic, and Composite Cardiovascular Burden

The elevated stroke risk documented in the meta-analysis cited above [25] has a demonstrable structural correlate in the cerebrovascular arterial wall. Carotid intima-media thickness is significantly greater in PA than in essential hypertension independently of blood pressure [183,189], and PA is associated with increased arterial stiffness and histologically confirmed carotid artery fibrosis relative to blood-pressure-matched controls [190,191,192]. These findings document a persistent vascular remodelling burden in PA that exceeds what haemodynamic load alone can account for; whether it contributes directly to the stroke excess in individual patients, or reflects one of several converging risk factors, cannot be established from observational data alone.

The diabetes excess documented in the meta-analysis [25] is further characterised by genotypic and hormonal patterning that implicates mechanisms beyond blood pressure. In the JPAS multicohort study of 2210 patients, diabetes prevalence was significantly higher in bilateral than unilateral PA and was independently associated with concurrent subclinical hypercortisolism, implicating both aldosterone excess and glucocorticoid co-secretion as additive diabetogenic signals [193]. Autonomous cortisol co-secretion occurs in a subset of patients with PA [194], and, in immune-lineage cells with limited 11β-HSD2 activity [195,196], co-secreted cortisol accesses the MR with affinity equivalent to that of aldosterone; whether this additional ligand burden amplifies downstream immune-lineage MR signalling and contributes independently to target-organ injury has not been prospectively evaluated. The molecular basis of aldosterone-driven insulin resistance in experimental systems involves ROS-mediated degradation of insulin receptor substrates IRS-1 and IRS-2 in adipocytes [197], disruption of adipokine signalling, and impairment of pancreatic beta-cell function [198,199,200]; the relative contribution of these mechanisms to insulin resistance in human PA has not been established in mechanistic human studies. Visceral adiposity amplifies this metabolic burden: in a prospective cohort, visceral adipose tissue volume was an independent predictor of new-onset diabetes in PA patients [201], consistent with convergence of aldosterone-driven insulin resistance, glucocorticoid co-secretion, and adiposity-related pathways rather than a single operative mechanism.

### 6.4. Therapeutic Implications of the Aldosterone–Immune Axis

The reconceptualisation of PA as a chronic fibro-inflammatory endocrine disorder rather than a purely haemodynamic disease has direct therapeutic consequences that extend beyond blood pressure management. The mechanistic evidence reviewed above highlights early diagnosis and timely definitive treatment as key determinants of structural disease regression. Delayed intervention may reduce the potential for complete reversal, as fibrogenic pathways can become progressively self-sustaining and trained haematopoietic progenitor populations may persist beyond biochemical cure. Consequently, adrenal vein sampling-guided treatment selection between surgical and medical management remains essential for identifying patients who may benefit from curative adrenalectomy and should be considered whenever surgery is a feasible option [22].

#### 6.4.1. Surgical Adrenalectomy

For biochemically confirmed unilateral PA attributable to a surgically resectable adenoma, laparoscopic adrenalectomy is the preferred definitive treatment. The Primary Aldosteronism Surgery Outcome international cohort confirmed biochemical cure of aldosterone excess in 94% of patients, with complete or partial blood pressure resolution in 84% [55]. These findings establish adrenalectomy as one of the most effective definitive treatments for secondary hypertension caused by unilateral PA. The superiority of surgery over medical therapy extends beyond biochemical correction to structural and immunological dimensions that are not captured by blood pressure or electrolyte endpoints. A retrospective cohort of 602 medically treated PA patients matched to 41,853 essential hypertension controls demonstrated a two-fold higher risk of myocardial infarction, heart failure hospitalisation, and stroke in medically treated PA despite equivalent blood pressure, establishing that medical therapy alone, even when biochemically adequate, does not eliminate the excess structural organ damage trajectory [186]. In contrast, adrenalectomy normalises long-term AF risk to essential hypertension-comparable levels at median 11.8-year follow-up [163], an outcome not achieved with inadequate medical therapy.

Beyond correcting autonomous aldosterone production, adrenalectomy eliminates the tumour itself, and, by extension, the associated intra-adrenal cellular microenvironment characterised by inflammatory cell infiltration [50]. Whether paracrine NF-κB, IL-6, and TNF-α signalling within this microenvironment contributes to *CYP11B2* expression through transcription factor-dependent routes not fully addressable by systemic MR antagonism remains uninvestigated in human APA tissue and constitutes a translational priority. The clinical importance of tumour removal is further reinforced by the duration-dependence of structural damage reversal. The duration of aldosterone excess prior to surgery is an independent predictor of the completeness of cardiovascular structural regression. In a prospective cohort of 54 PA patients followed for a mean of 6.4 years, left ventricular mass index declined significantly within the first year only in adrenalectomised patients [202], and post-treatment eGFR decline directly unmasked the extent of pre-existing structural nephron loss in proportion to preoperative disease duration [188]. These findings provide the mechanistic rationale for the absolute clinical priority of early diagnosis.

#### 6.4.2. Steroidal Mineralocorticoid Receptor Antagonists and Renin-Guided Therapy

For bilateral adrenal hyperplasia, surgically inoperable patients, or those who decline surgery, MR antagonist therapy with spironolactone or eplerenone constitutes the established medical management. Spironolactone is a steroidal non-selective MR antagonist with anti-androgenic and progesterone receptor cross-reactivity that limits tolerability, particularly in male patients; eplerenone is more receptor-selective with reduced off-target endocrine effects at the cost of lower MR binding affinity and higher required dosing [41,203,204]. The clinical evidence base for spironolactone in resistant hypertension was established by the PATHWAY-2 trial, which demonstrated spironolactone as the superior fourth-line antihypertensive agent, outperforming bisoprolol and doxazosin in patients with confirmed treatment-resistant hypertension [42]. Restoration of detectable renin activity, rather than blood pressure normalisation alone, represents the most appropriate pharmacodynamic indicator of adequate MR blockade [22]. Inadequate MR blockade, defined operationally by persistent renin suppression despite MRA therapy, is independently associated with significantly elevated residual AF risk (adjusted HR 2.55; 95% CI 1.75–3.71) compared with essential hypertension controls [164], supporting renin activity restoration as the more clinically informative pharmacodynamic endpoint than blood pressure normalisation alone. Whether complete renin activity restoration is sufficient to silence the trained innate immunity programme established during aldosterone excess, and whether it reverses bone marrow haematopoietic myeloid dominance, has not been directly investigated and remains an important unanswered translational question that warrants investigation in prospective PA-specific cohorts incorporating longitudinal immunological phenotyping.

## 7. Non-Steroidal Mineralocorticoid Receptor Antagonists

Non-steroidal MR antagonists such as finerenone, a third-generation non-steroidal MR antagonist with substantially higher receptor selectivity, a distinct tissue distribution profile favouring cardiac and renal compartments, and not clinically meaningful cross-reactivity at androgen or progesterone receptors, confer a pharmacological and tolerability profile distinct from both spironolactone and eplerenone [203]. Finerenone demonstrated significant reductions in composite renal and cardiovascular outcomes in FIDELIO-DKD [205], cardiovascular events in FIGARO-DKD [206] and worsening heart failure in FINEARTS-HF [207,208] at a magnitude exceeding blood pressure reduction alone. A pilot open-label randomised controlled study by Hu and colleagues, directly comparing finerenone and spironolactone in patients with PA, demonstrated comparable blood pressure-lowering efficacy between the two agents, with finerenone associated with a smaller increment in serum potassium despite similar mean daily doses [209]. The apparently greater antihypertensive response observed with finerenone in PA [210], compared with its more modest blood pressure effects in FIDELIO-DKD and FIGARO-DKD, most likely reflects the distinct endocrine pathophysiology of PA rather than intrinsic differences in drug efficacy [205,207].

Esaxerenone, currently approved only in Japan, carries a progressively expanding clinical evidence base across multiple hypertensive phenotypes [211]. It has demonstrated antihypertensive efficacy in essential hypertension (ESAX-HTN trial) [212], blood pressure reduction and urinary albumin suppression in diabetic kidney disease (ESAX-DN and EX-DKD trials) [213,214], left ventricular mass index regression in hypertensive patients with left ventricular hypertrophy (ESES-LVH trial) [215], blood pressure reduction in nocturnal hypertension among patients with resistant hypertension (EARLY-NH trial) [216], and non-inferiority to trichloromethiazide in head-to-head antihypertensive comparison (EXCITE-HT trial) [217]. In PA specifically, a prospective study of 25 PA patients demonstrated significant reductions in urinary albumin-to-creatinine ratio and NT-proBNP independent of blood pressure reduction, alongside quality-of-life improvement at six months [218]. A retrospective cohort of 87 PA patients confirmed systolic blood pressure reduction, potassium restoration, and elimination of aldosterone-driven glomerular hyperfiltration without treatment discontinuation due to hyperkalaemia or renal dysfunction [219]. In preclinical murine aldosterone infusion models, esaxerenone additionally suppressed renal M1 macrophage proliferation through the MR/*CSF1* axis and macrophage-to-myofibroblast transition through the MR/TGF-β1 axis [86,151]. These preclinical findings suggest that esaxerenone may confer anti-inflammatory and anti-fibrotic organ protection beyond blood pressure reduction, although this hypothesis has yet to be evaluated in prospective randomised studies of patients with PA.

### Immunological Endpoints in Treatment Monitoring

The current clinical framework for PA treatment response monitoring centres on aldosterone-to-renin ratio normalisation, blood pressure control, and electrolyte correction. Although this framework adequately assesses the hormonal and haemodynamic consequences of treatment, it does not directly evaluate resolution of the underlying immune and fibrotic processes increasingly recognised as contributors to long-term organ damage. The evidence reviewed here identifies a biologically grounded set of candidate immunological endpoints that, if prospectively validated in adequately powered PA cohorts with paired pre-treatment and post-treatment sampling, would substantially advance the precision of treatment response assessment beyond conventional biochemical markers. The monocyte-to-lymphocyte ratio and neutrophil-to-lymphocyte ratio are obtainable from routine full blood counts, are elevated in PA in proportion to plasma aldosterone concentration [159]; however, these inflammatory indices remain unchanged in the acute phase (one week) following adrenalectomy, with baseline MLR instead serving as a predictor of long-term clinical success [220]. Their practical advantage is accessibility without specialist assay infrastructure, making them immediately translatable to clinical monitoring protocols. The circulating Th17/Treg ratio, documented as significantly imbalanced in human PA relative to blood-pressure-matched resistant hypertension controls [54], is quantifiable by standard flow cytometry and provides a direct immunophenotypic read on the adaptive immune dysregulation that conventional markers do not capture.

Trained macrophage cytokine production capacity, assessed by ex vivo oxLDL or TLR-ligand stimulation of patient-derived monocyte-derived macrophages [85], captures the epigenetic dimension of the aldosterone-driven inflammatory programme at the tissue differentiation stage where it is operative, consistent with the established methodology for assessing trained immunity status in clinical cohorts [221]. Because this phenotype is invisible to circulating inflammatory biomarker panels, it represents a monitoring endpoint specifically sensitive to the trained immunity component of PA-associated immune dysregulation that biochemical cure alone may not fully resolve. Bone marrow 18F-FDG uptake on positron emission tomography characterises haematopoietic myeloid activation at the progenitor level [52], and could serve as an imaging biomarker of post-cure bone marrow normalisation, directly addressing the question of whether trained progenitor pool replacement follows the temporal trajectory of clinical risk reduction after treatment. Galectin-3, a macrophage-derived mediator of fibroblast activation established as a downstream effector of aldosterone-driven vascular and cardiac fibrosis [222,223], represents a candidate circulating biomarker of the macrophage–fibroblast fibrogenic axis whose normalisation post-treatment could serve as a surrogate for structural fibrosis regression independently of left ventricular mass, addressing the known dissociation between mass regression and diastolic function recovery that echocardiographic endpoints alone cannot resolve. Prospective validation of these candidate immunological biomarkers in adequately powered PA-specific clinical studies could refine the definition of complete treatment response beyond conventional biochemical cure, identify patients with persistent inflammatory activity who may benefit from closer surveillance or adjunctive therapy, and establish a biological framework for evaluating future immune-targeted interventions in PA.

## 8. Conclusions

The accumulated evidence supports a fundamental reconceptualisation of PA: rather than simply an endocrine cause of hypertension, it is increasingly recognised as a chronic fibro-inflammatory disorder in which autonomous mineralocorticoid excess engages immune-effector pathways that contribute to cardiovascular, renal, and metabolic injury beyond the effects of blood pressure elevation alone. Human studies consistently demonstrate a disproportionate burden of cardiovascular events compared with haemodynamically matched controls, together with monocyte transcriptomic reprogramming; Th17/Treg imbalance not replicated in haemodynamically matched essential hypertension controls, suggesting a specific relationship with aldosterone excess rather than hypertension per se; and evidence of ex vivo trained innate immunity.

Mechanistic studies in experimental systems have further established roles for M1 macrophage polarisation, macrophage-to-myofibroblast transition, dendritic cell-mediated adaptive immune dysregulation, and haematopoietic progenitor epigenetic reprogramming. While these findings are biologically coherent with observations in human PA, direct confirmation within human tissues remains an important translational priority. Equally, several critical questions remain unresolved and require prospective human investigation, including whether trained immunity persists following biochemical cure, whether residual cardiovascular risk reflects the lifespan of trained haematopoietic progenitor populations, whether immune remodelling differs according to somatic mutational subtype, and whether immunological biomarkers can serve as precision indicators of treatment response.

These mechanistic insights also have important therapeutic implications. Early diagnosis and timely intervention maximise the potential for structural reversibility before fibrogenic pathways become progressively self-sustaining. Surgical adrenalectomy eliminates both the source of autonomous aldosterone production and the associated intra-adrenal inflammatory microenvironment, whereas MR antagonists, titrated to restoration of renin activity, target the downstream consequences of persistent receptor activation in bilateral disease.

In summary, PA should be recognised not only as an endocrine disorder of autonomous aldosterone excess but also as a systemic fibro-inflammatory disease in which immune dysregulation contributes substantially to long-term organ damage. Future management strategies should therefore extend beyond achieving biochemical and haemodynamic control to encompass suppression of the aldosterone–immune axis through earlier diagnosis, appropriate selection of definitive or medical therapy, and the incorporation of immunologically informed biomarkers to guide risk stratification and treatment monitoring.

## Data Availability

No new data were created or analyzed in this study.

## Abbreviations

11β-HSD2	11β-hydroxysteroid dehydrogenase type 2
AF	Atrial fibrillation
AngII	Angiotensin II
APA	Aldosterone-producing adenoma
CAMK	Calcium/calmodulin-dependent protein kinase
GR	Glucocorticoid receptor
HSPC	Haematopoietic stem and progenitor cell
MR	Mineralocorticoid receptor
NO	Nitric oxide
PA	Primary aldosteronism
RAAS	Renin–angiotensin–aldosterone system
ROS	Reactive oxygen species
TGF-β1	Transforming growth factor beta 1
Th17	T helper 17
VSMCs	Vascular smooth muscle cells
ZG	Zona glomerulosa
